# *MYCN* amplification predicts poor prognosis based on interphase fluorescence in situ hybridization analysis of bone marrow cells in bone marrow metastases of neuroblastoma

**DOI:** 10.1186/s12935-017-0412-z

**Published:** 2017-03-31

**Authors:** Zhi-Xia Yue, Cheng Huang, Chao Gao, Tian-Yu Xing, Shu-Guang Liu, Xing-Jun Li, Qian Zhao, Xi-Si Wang, Wen Zhao, Mei Jin, Xiao-Li Ma

**Affiliations:** Beijing Key Laboratory of Pediatric Hematology Oncology, National Key Discipline of Pediatrics, Ministry of Education, MOE Key Laboratory of Major Diseases in Children, Hematology Oncology Center, Beijing Children’s Hospital, Capital Medical University, 56 Nanlishi Road, Beijing, 100045 China

**Keywords:** Neuroblastoma, *MYCN* amplification, Fluorescence in situ hybridization, Bone marrow metastasis

## Abstract

**Background:**

*MYCN* gene amplification is related to risk stratification. Therefore it is important to identify accurately the level of the *MYCN* gene as early as possible in neuroblastoma (NB); however, for patients with bone marrow (BM) metastasis who need chemotherapy before surgery, timely detection of the *MYCN* gene is not possible due to the unavailability of primary tumors.

**Methods:**

*MYCN* gene status was evaluated in 81 BM metastases of NB by interphase fluorescence in situ hybridization (FISH) analysis of BM cells. The clinicobiological characteristics and prognostic impact of *MYCN* amplification in NB metastatic to BM were analyzed.

**Results:**

*MYCN* amplification was found in 16% of patients with metastases, and the results were consistent with the primary tumors detected by pathological tissue FISH. *MYCN* amplification was associated with age, lactate dehydrogenase (LDH) levels and prognosis (*P* = 0.038, *P* < 0.001, *P* = 0.026). Clinical outcome was poorer in patients with *MYCN* amplification than in those without amplification (3-year EFS 28.8 ± 13.1 vs. 69.7 ± 5.7%, *P* = 0.005; 3-year OS 41.5 ± 14.7 vs. 76.7 ± 5.5%, *P* = 0.005).

**Conclusions:**

*MYCN* amplification predicts a poor outcome in NB metastatic to BM, and interphase FISH of bone marrow cells provides a timely direct and valid method to evaluate the *MYCN* gene status.

## Background

Neuroblastoma (NB),derived from the postganglionic sympathetic nervous system, is the most common extracranial malignancy in children [1]. It represents 8–10% of pediatric tumors and accounts for >10% of all pediatric cancer mortality [2]. Early NB often is clinically unrecognized [3]. The primary tumor usually occurs in the abdomen (60%), but more than 50% of children with neuroblastoma present with metastasis at diagnosis, and bone marrow (BM) is the most common site of metastasis (70.5%) [4]. In recent years, substantial progress has been made in the treatment effect of NB combinations of chemotherapy, radiotherapy, surgical resection and hematopoietic stem cell transplantation; however the 5-year overall survival (OS) is still less than 50% in high-risk NB [5, 6].

The *MYCN* gene codes for an oncogenic transcription factor and belongs to the *MYC* family of genes [7]. Amplification of the *MYCN* gene was observed in approximately 20% of all NB, and is considered to be a molecular marker to identify high-risk patients [8]. Because *MYCN* gene amplification is related to risk stratification, it is therefore important to identify accurately the level of *MYCN* gene amplification as early as possible in order to avoid either under- or over-treating patients [9–11]. At present, it is imperative to detect *MYCN* gene amplification in many established NB treatment protocols by fluorescence in situ hybridization (FISH) [12]. The samples are usually tissue from primary tumors; however, for those patients who need chemotherapy prior to surgery, timely identification of the level of *MYCN* gene is not possible because of the limited nature of tumor biopsies. BM is the most common site of metastasis, and there are no reports on the status of the *MYCN* gene in BM cells in patients with NB metastatic to BM.

The aim of our study was to clarify the clinical value of interphase FISH technique in detecting *MYCN* amplification of BM cells and evaluate the biological characteristics and prognostic impact of *MYCN* status in NB metastatic to BM.

## Methods

### Patients and treatment

A total of 81 pediatric patients aged 5–103 months (median 39 months) with newly diagnosed NB with BM metastasis at the Haematology Oncology Centre of Beijing Children’s Hospital, Capital Medical University, between January 2012 and August 2014 were enrolled in this research. The criterion for patient inclusion was ≥20% NB cells in diagnostic BM samples. BM samples of all patients were collected at diagnosis. Cells were cultured in RPMI-1640 medium supplemented with 20% fetal bovine serum. After 24 h in culture, the cells were treated with 0.075 mol/LKCl for 30 min, and then were fixed twice in a 3:1 mixture of methanol:acetic acid. Then the cells were stored in the above fixative solution at 4 °C.

The BCH-NB-2007 protocol [13] was used in all patients. All patients were followed until June 2016, with a median follow-up time of 28.2 months (range 0.5–54 months). This research and the BCH-NB-2007 protocol were approved by the Beijing Children’s Hospital Institutional Ethics Committee. Informed consent was obtained from the parents or guardians of each patient according to the Declaration of Helsinki.

### Morphologic analysis and diagnostic biomarkers detection

Microscopic examinations of BM aspirates and biopsies were performed to determine the presence of NB cells. The presence of NB cells was determined by 2 independent laboratory pathologists, expert in this area. Serum tumour markers such as lactate dehydrogenase (LDH) and neuron specific enolase (NSE) levels were detected at the time of diagnosis in all patients. Urinary homovanillic acid (HVA) and vanillylmandelic acid (VMA) are typically measured in urine for the diagnosis and monitoring of NB.

### FISH analysis of bone marrow cells

The FISH technique was applied using a DNA probe LSI *N*-*MYC* (2p24)/CEP2 (2p11.1-q11.1) Dual Color Probe (Vysis) to count the number of *MYCN* copies in relation to the number of chromosomes 2. FISH was performed in a dual-color procedure following the manufacturers’ instructions. BM cells fixed in methanol-acetic acid solution were dropped onto cleaned glass slides. The slides were denatured in 70% formamide/2× standard saline citrate (SSC) at 73 °C for 5 min, then dehydrated through an ethanol series of 70, 85 and 100% for 2 min, each. The probe mixture contained 7 μl hybridization buffer, 1 μl probe and 2 μl double-distilled water. The mixture was denatured for 5 min at 73 °C, and then applied on the slide. Hybridization was performed overnight at 37 °C under sealed coverslips. The slide was washed immediately in 0.4 × SSC/0.3%NP-40 (PH7.5) at 73 °C for 1 min, followed by 2 × SSC/0.1%NP-40 (PH7.0) at room temperature for 1 min. The nuclei were counterstained with 10 μl DAPI, and 400 cells were counted on one slide. Fluorescence images were taken with a Leica DM6000B microscope.

In accordance with the recommendations of the European Neuroblastoma Quality Assessment group [14], amplification was defined as a >fourfold increase of *MYCN* signals in relation to the number of chromosome 2. In addition, a 1.5- to 4-fold 2p24 copy number in relation to the copy number of chromosome 2 is defined as 2p24 gain, which could reflect either gain of 2p or gain of the *MYCN* gene. Additionally, the status of 1p36 and 11q were determined with DNA probes from Kreatech [ON1q21/SRD(1p36)] and Vysis [LSI MLL Dual Color, Break Apart Rearrangement Probe] respectively.

### Statistical analysis

June 30, 2016 was chosen as the reference date for the end of data collection for purposes of statistical analysis. Comparisons between study groups with or without *MYCN* amplification and associations between patient pretreatment characteristics were evaluated by nonparametric tests. Event-free survival (EFS) was defined from the date of diagnosis to the date of the first event (for example, relapse or death), or the last contact with patients in continuous CR. Overall survival (OS) was defined as the time from the diagnostic date through the date of death (from any cause), or the last contact with patients in continuous CR. EFS and OS distribution with or without *MYCN* amplification was estimated with the Kaplan–Meier survival analysis. All tests were two-sided with a *P* < 0.05 considered statistically significant. SPSS 16.0 software was used for all statistical analyses.

## Results

### Clinical and laboratory characteristics of patients with BM metastases of NB

The criteria for diagnosis and staging adhered to the international neuroblastoma staging system (INSS). We divided these patients into four groups according to age (<12, 12–18, 18–24, >24 months). Among the 81 cases of NB metastatic to BM, there were significantly more males (63%) than females (37%) with a ratio 1.7:1.

All 81 patients had stage 4 disease. Patients were stratified into 3 risk groups (low-risk, LR; intermediate-risk, IR; high-risk, HR). *MYCN* amplification was seen in 13 patients (16.0%). Twenty-six patients (31.1%) had very high serum LDH levels (≥1500 IU/L), and 71 patients (87.7%) had very high serum NSE levels (≥100 UG/L).The median follow-up time was 21.4 months (range 0.5–44.0). Twenty-seven patients (33.3%) suffered relapse or died during the follow-up period. The main clinical characteristics of the 81 patients are shown in Table 1.

**Table 1:** Baseline characteristics of patients with metastatic NB (N = 81)

Characteristics	Total n (%)
Age (months)	
<12	3 (3.7)
12–18	4 (4.9)
18–24	11 (13.6)
>24	63 (77.8)
Gender	
Male	51 (63.0)
Female	30 (37.0)
Treatment group	
LR	3 (3.7)
IR	3 (3.7)
HR	75 (92.6)
Clinical stage	
IV	81 (100)
*MYCN* gene	
Amplification	13 (16.0)
Normal	68 (84.0)
LDH (IU/L)	
<1500	55 (67.9)
≥1500	26 (31.1)
NSE (UG/L)	
<100	10 (12.3)
≥100	71 (87.7)
Primary site	
Abdomen	73 (90.1)
Thorax	6 (7.4)
Others	2 (2.5)
Event	
No event	54 (66.7)
Relapse	20 (24.7)
Death	7 (8.6)

*NB* neuroblastoma, *LR* low-risk, *IR* intermediate-risk, *HR* high-risk, *LDH* lactate dehydrogenase, *NSE* neuron specific enolase

### *MYCN*-status and other genetic markers of bone marrow cells by interphase FISH

**Fig. 1:**
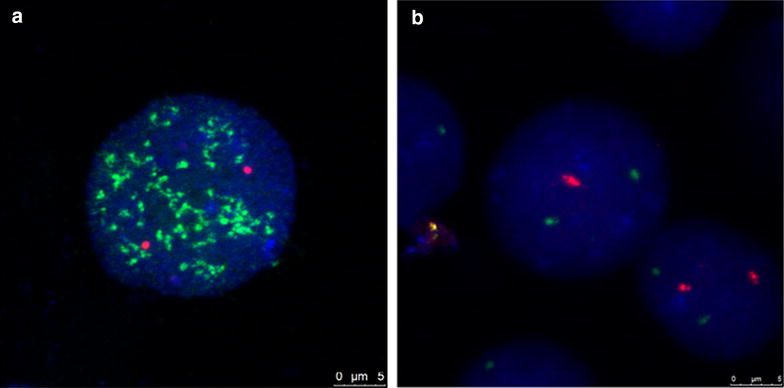
Representative FISH images of BM cells. **a** The status of *MYCN* amplification using a dual-color probe. *Green signals* represent the specific probe for *MYCN* and *red signals* stand for centromeric chromosome 2 probes. *MYCN* signals show more than 30 copies within the nuclei. **b** The status of 1pLOH using a dual-color probe. *Green signals* represent the copy numbers at 1q21 and *red signals* are the specific DNA probe of 1p36. *Bars* 5 μm

We investigated the *MYCN* status in all patients and amplification was seen in 13 cases (16.0%; Fig. 1). The signal copy numbers of *MYCN* gene were >15 copies, and the positive cells accounted for more than 80% in every case. An additional six cases (7%) displayed *MYCN*-gain with the signal copy numbers between 2 and 9 copies. Loss of heterozygosity (LOH) of 1p36 and 11q deletion were also analyzed in the *MYCN*-amplified cases by interphase FISH. Eight patients (61.5%) presented *MYCN* amplification and 1pLOH simultaneously; however, no 11q deletion was detected in the *MYCN*-amplified NB. The clinical characteristics and FISH results of 13cases are presented in Table 2.

**Table 2:** Overview of FISH results and patient clinical characteristics in *MYCN*-amplified NB

	Number	Gender		Age		LDH		NSE	
		Male	Female	12–24	>24	<1500	≥1500	<100	≥100
1pLOH									
(+)	8	5	3	6	2	0	8	0	8
(−)	5	2	3	0	5	3	2	0	5
MLL									
(+)	0	0	0	0	0	0	0	0	0
(−)	13	7	6	6	7	3	10	0	13

*NB* neuroblastoma, *LDH* lactate dehydrogenase, *NSE* neuron specific enolase

In this study, the primary tumors of 53 patients were also detected by pathological tissue FISH, and *MYCN* amplification was seen in 14 cases. Only one case had a different result, with the primary tumor sample showing *MYCN* amplification, but the BM cell sample showing normal signals. The remaining other 42 cases showed consistent results in the two different specimens.

### Impact of *MYCN* amplification on prognosis of NB metastatic to BM

**Fig. 2:**
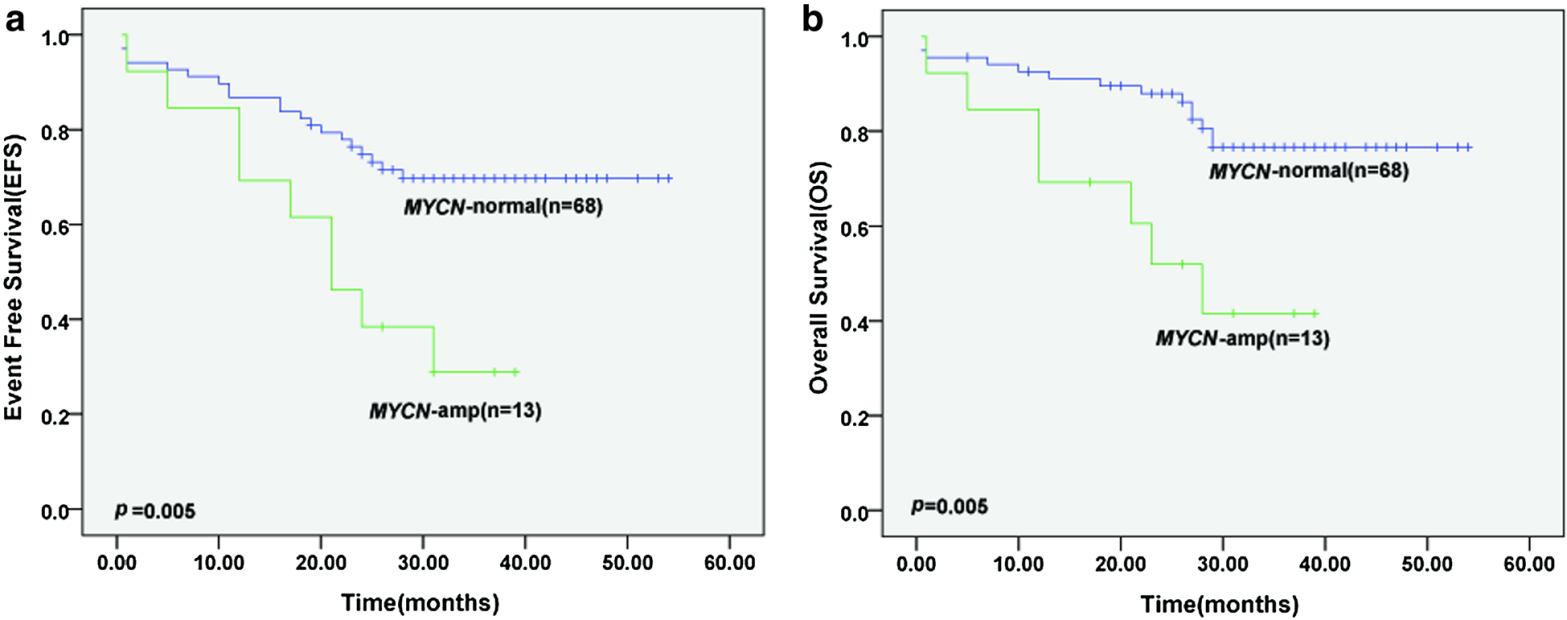
The prognostic significance of MYCN amplification in bone marrow metastatic NB. **a**, **b** Event free survival (EFS) and overall survival (OS) of 81 patients stratified by MYCN status

The 3-year EFS rate of the 81 patients was 60.8 ± 7.0%, with a median follow-up of 21.4 months. To evaluate the prognostic value of *MYCN* gene in NB metastatic to BM, we divided the whole cohort of 81 patients into *MYCN*-normal and *MYCN*-amplification groups (68 and 13 cases, respectively). During the follow-up period, more patients suffered from relapse or died in *MYCN*-amplification group (8/13, 61.5%) than in the *MYCN*-normal group (19/68, 27.9%). Clinical outcome was poorer in patients with *MYCN* gene amplification than in those without amplification (3-year EFS 28.8 ± 13.1 vs. 69.7 ± 5.7%, *P* = 0.005; 3-year OS 41.5 ± 14.7 vs. 76.7 ± 5.5%, *P* = 0.005; Fig. 2).

### Relationship of *MYCN* status to clinicobiological characteristics of NB metastatic to BM

We next assessed the associations of *MYCN* status at diagnosis with the clinicobiological characteristics of the 81 cases of NB metastatic to BM. We divided these patients into two groups according to the levels of *MYCN*. We observed a significant association between *MYCN* amplification and age, LDH levels as well as prognosis (*P* = 0.038, *P* < 0.001, *P* = 0.026). No significant correlations between *MYCN* amplification and gender, treatment, NSE levels, or primary site were apparent; however, we found that all 13 cases of *MYCN* amplification occurred in patients in the high-risk (HR) treatment group, high serum NSE levels group, and in those whose primary site was mainly in the abdomen, although the difference was not statistically significant.

In this study, *MYCN* amplification was found in 16% of NB metastatic to BM and was found in different proportions of children of different ages. Children younger than 1 year had the best results with no amplification found. Children older than 2 years exhibited the highest prevalence of *MYCN* amplification (53.8%) compared with any other group. Among patients between 18 and 24 months of age, 30.8% had *MYCN* amplification as opposed to 15.4% of patients between 12 and 18 months of age (Table 3).

**Table 3:** Relationship of *MYCN* status to the clinicobiological characteristics of BM metastatic NB (n = 81)

Characteristics	Total n (%)	*MYCN*-amplification n (%)	*MYCN*-normal n (%)	*P* values*
Gender				
Male	51 (63.0)	7 (53.8)	44 (64.7)	0.536
Female	30 (37.0)	6 (46.2)	24 (35.3)	
Age (months)				
<12	3 (3.7)	0 (0)	3 (4.4)	0.038
12–18	4 (4.9)	2 (15.4)	2 (2.9)	
18–24	11 (13.6)	4 (30.8)	7 (10.3)	
>24	63 (77.8)	7 (53.8)	56 (82.4)	
Treatment group				
LR	3 (3.7)	0 (0)	3 (4.4)	0.999
IR	3 (3.7)	0 (0)	3 (4.4)	
HR	75 (92.6)	13 (100)	62 (91.2)	
LDH (IU/L)				
<1500	55 (67.9)	3 (23.1)	52 (76.5)	<0.001
≥1500	26 (31.1)	10 (76.9)	16 (23.5)	
NSE (UG/L)				
<100	10 (12.3)	0 (0)	10 (14.7)	0.352
≥100	71 (87.7)	13 (100)	58 (85.3)	
Primary site				
Abdomen	73 (90.1)	12 (92.3)	61 (89.7)	0.999
Thorax	6 (7.4)	1 (7.7)	5 (7.4)	
Others	2 (2.5)	0 (0)	2 (2.9)	
Event				
No event	54 (66.7)	5 (38.5)	49 (72.1)	0.026
Event	27 (33.3)	8 (61.5)	19 (27.9)	

*NB* neuroblastoma, *BM* bone marrow, *LR* low-risk, *IR* intermediate-risk, *HR* high-risk, *LDH* lactate dehydrogenase, *NSE* neuron specific enolase

* Data were calculated by χ2 test

## Discussion

Amplification of the *MYCN* proto-oncogene is a powerful prognostic indicator of advanced stages and rapid tumor progression [15]. In our clinical practice, some patients suffered from treatment failure mainly because of incorrect risk stratification [16]. For those patients who need chemotherapy before surgery, timely assessment of the level of *MYCN* gene expression may not be possible due to the lack of availability of primary tumors. In addition, the results of *MYCN* gene of primary tumors maybe inaccurate after toxic chemotherapy, leading to insufficient dosage and period of treatment. In this study, we found interphase FISH of BM cells was reliable in detecting *MYCN* gene status in BM metastases of NB. In our study, *MYCN* amplification was present in 16.0% of patients in whom the positive cells accounted for more than 80% of BM cells, which was close to the percentage reported by other groups [17, 18]. In addition, we obtained nearly consistent results in the two different specimens including BM cells and primary tumors. Interphase FISH analysis provides a direct and rapid method. Only a small number of tumor cells are required, compared with primary tumor biopsies, and FISH is more sensitive, counting the *MYCN* copy number in each single cell.

A total of 81 patients with BM metastasis were enrolled in this research. The median age was 39 months (range 5–103 months) with 91.4% of patients older than 18 months. In our study, there were significantly more males (63%) than females (37%), with a ratio of 1.7:1, which is higher than the proportion reported by other groups [19]. All patients had stage 4 disease, and 92.6% of cases belonged to the HR group. This is consistent with the International Neuroblastoma Risk Group (INRG) suggestion that an age-at-diagnosis cutoff of greater than 18 months is associated with higher risk disease [20]. We found abdomen to be the most common site (90.1%); thorax was the second (7.4%), and only one NB primary was located in the epidural area, and one in the sacroiliac region.

Patient age is a clinical variable with independent prognostic value in NB [21]. In this study, the prevalence of *MYCN* amplification displayed obvious association with age. The prevalence reached a peak of 53.8% in patients older than 2 years, was 30.8% for those between 18 and 24 months of age, and 15.4% in patients 12–18 months of age. No amplification was found in patients younger than 1 year. These results demonstrated that in NB metastases in BM, *MYCN* amplification is the age-dependent variation.

We found that amplification of the *MYCN* oncogene was significantly correlated with poor EFS and OS in our study, which is consistent with previous findings [22]. Serum markers, such as lactic dehydrogenase (LDH), and neuron-specific enolase (NSE) were recognized as prognostic markers especially in metastatic NB [23]. In this study, we found that 76.9% (10/13) of patients with *MYCN*-amplification had elevated serum LDH levels, in contrast to 23.1% (3/13) of those without *MYCN* amplification. This difference was statistically significant (*P* < 0.001); however, there was no significant association between *MYCN* amplification and NSE levels even though all patients with *MYCN* amplification had high serum NSE levels. This phenomenon might reflect the fact that LDH was a better clinical prognostic marker than NSE for patients with NB metastatic to BM.

1pLOH and loss of 11q occur in approximately 25–30% of primary NB, and are both associated with poor outcome [24]. Recent studies demonstrated that *MYCN* is a direct target of miR-34a, which maps to the 1p36 region, and 1pLOH results in loss of miR-34a, which may lead to amplification of *MYCN* gene [25]; however, the correlation of *MYCN* and 1pLOH remains controversial among different groups [26]. In our study, 61.5% of NB with *MYCN*-amplification had 1pLOH suggesting that a positive relationship maybe exist. No 11q deletion was detected in the *MYCN*-amplified NB in this study. This was consistent with the previous finding of an inverse relationship between *MYCN* amplification and 11q loss [27]. Because we did not detect the two markers in the absence of *MYCN* amplification, we can not make a statistical analysis.

## Conclusions

To date, this is the largest study of Chinese pediatric patients with NB metastatic to BM treated in a single institution. Our focus was on long-term outcomes in patients with or without *MYCN* amplification detected by interphase FISH of bone marrow cells. Our study demonstrated an association of *MYCN* amplification with poor long-term prognosis in patients with NB metastatic to BM. In our study, interphase FISH of bone marrow cells provided a direct and valid method to assess *MYCN* amplification and 1pLOH.

## Abbreviations

NB: neuroblastoma
FISH: fluorescence in situ hybridization
LDH: lactate dehydrogenase
NSE: neuron specific enolase
EFS: event-free survival
OS: overall survival
